# Notes and News

**Published:** 1892-08-20

**Authors:** 


### Notes and News.

COLLECTED AND COMMUNICATED.

The Monkwearmouth and Southwick Hospital has at length been freed from a very objectionable thorn in its side. The Smithson Square property, which was purchased last year, has been demolished, and with it the drunken brawls which made its reputation one of unenviable notoriety. These brawls were not the only blots in the charming locality which has gone to its rest, there were sanitary dangers which threatened the hospital and which now happily are no more. The open space where the houses formerly stood will no doubt prove a pleasant recreation ground for convalescent patients. A ward for the treatment of female cases is urgently needed, and it is satisfactory to know that the Committee recognise the need, though unfortunately want of funds appears to stand in the way of making the desired provision. The ambulance classes at the hospital do not seem to be as popular as could be wished amongst working men and their wives, who are slow to recognise the value of such training.

The isolation block in connection with the Sunderland Infirmary has now been completed, and it is expected that it will be opened formally by Miss Forster, who has defrayed the cost of the building. This will probably prevent a recurrence of the falling off in numbers of patients treated during the past financial year, which was due to outbreaks of infectious diseases in the infirmary, which in future will be provided for in the isolation wards. Activity has also been shown in another direction. The need of a convalescent home in connection with the infirmary, which has long been pressing, has been met through the generosity of the Victoria Hall Distress Fund Committee, who have handed to the infirmary board a sum of £4,000, with which they have been enabled to purchase and adapt for the reception of convalescent patients Heatherdene House, Harrogate. The increased usefulness of the institution merits generous support, which has recently fallen somewhat in arrear owing to the disturbances in the industrial world. Now these are over, at least for the present, no doubt subscriptions will again come in more steadily.

The example of the Northumberland Miners' Association, which has increased its subscription to the Newcastle Royal Infirmary from £150 to £200 a year, should be noted by the corresponding association in Durham. This latter at present contributes no money, but a very large number of patients; it cannot therefore gracefully decline to respond handsomely to the call which is going to be made on it. The question of the memorial wing to be added in memory of Dr. Collingwood Bruce has called forth much difference of opinion as to the desirability of a new site, and the matter will not be decided definitely for another month.

The Meath Home for Epileptics is now ready, and before the opening upwards of one hundred applications for admission have already been received. The Duchess of Albany, ever ready to be kind and helpful, went down to Godalming, and under the happiest circumstances declared the home to be open. Now the subscriptions are the principal things required to perfect the scheme, about which so much has been said both for and against. We quite agree that a guarantee of skilled treatment ought to be forthcoming, the supervision of nurses will not be sufficient, and the nurses, moreover, ought most assuredly to be "mental" nurses, But if the money can be extracted from the public pocket, and by judicious management this is generally possible, all these matters can be attended to. Lady Meath has the welfare of epileptics at heart, or she would not do as much as she has done.

The reasons against the proposed extension of the Manchester Royal Infirmary on the present site, to which we alluded in our last issue, have been set forth in a memorandum which has been issued to all the Infirmary Trustees. They put in a clear light the grounds of the opposition which has been daily gaining strength to the policy advocated by the Board of Management. Only those who live in or have visited Manchester in the autumn or winter when enveloped in one of its delightful fogs, which almost make London blush for her reputation in this direction, can fully appreciate the objections against the collection of an increased number of sick persons in the locality in question. The traffic in Piccadilly makes it one of the noisiest thoroughfares in the city, and patients who are seriously ill are already much troubled by the unceasing din of the traffic. Under the proposed scheme the additions to the Infirmary will face Piccadilly, and so add considerably to the distress of the patients. Equally strong objections can be alleged against the scheme on the ground that the population is yearly increasing in the outskirts of the city, whereas it is decreasing in the centre, and a hospital which is not easily accessible to the population it is supposed to serve loses more than half its value. It is of no small consideration that a crowded portion of the town should not be made still more crowded; this consideration alone makes any further encroachment on the site undesirable. It is also asserted, not without strong show of reason, that the proposed scheme is totally inadequate to meet the needs of the district. Any extension should certainly be on a scale sufficiently large to make the possibility of raising the question of further extension in a few years' time very remote.

The chair at the Quarterly Court of Governors of the Hospital for Consumption, Brompton, was taken by Mr. T. P. Beckwith. The report stated that the benefits of the hospital had continued to be abundantly sought after and valued. The 321 beds in the two buildings had remained fully occupied; and besides the treatment in the wards, 130 patients had been sent to the Seaside Convalescent Home at Sandgate, and maintained there at the entire expense of the hospital. In addition to the periodical cleaning, &c., a large amount of internal painting is now needed. This will involve very considerable outlay.

---

It has been said of women, that without them the beginning of life would be helpless, its middle without joy, and its end without consolation. Scientists are not so kind in their valuation of the fair sex. An American professor holds that women develop early, but early cease to grow mentally, and an Italian professor is asserted to be guilty of pronouncing women to be less sensitive than men, and as regards their receptive and perceptive organs to represent an incompletely developed type. But in spite of the dogmatisms of professors, women are holding their own in the mental, and for that matter in the physical, world, for the first ascent of the Matterhorn this season was made by a lady.

---

Those who are interested in the work of the Royal Sea Bathing Infirmary for Scrofula at Margate, and it is much to be wished more people had the welfare of the institution at heart, are rightly anxious as to its future. The down-hill course of its finances has been painfully steady of recent years, and its position can best be explained by stating that the hospital which is capable of accommodating 220 patients has reduced the number to 80. At the recent annual meeting it was asserted that one hundred people were then waiting for admission—a number which is daily increasing. If it is correct that patients are frequently sent to the Infirmary who are absolutely incurable, it appears that an unjust tax is placed thereby on the resources of the institution, which is not and never was designed for such cases. It is certainly most undesirable, from a financial point of view alone, that such cases should be accepted. Inasmuch as the institution is a national one, the support should be forthcoming necessary to enable the management to extend its operations to their full scope.

---

It is quite clear that in the event of a severe outbreak of fever in London the resources of the Asylums Board are nothing like sufficient to meet it, and if other localities are going to follow Tottenham's example, the lines of the Board will not lie in pleasant places. Where we shall ultimately find a resting-place for our fever hospitals in the future it will take a far-seeing mind to decide. Tottenham, its Public Health Association and its thousands of inhabitants are up in arms at the prospect of the Local Government Board commencing operations on the proposed site. Political changes, one householder hopes, may put a veto on the decision of the Tory Secretary to create a "plague spot" in Tottenham, and he goes so far as to suggest that the Liberal Secretary shall revoke the order. Eighty-five patients were admitted into the fever hospitals on Monday, and accommodation is becoming a serious matter. If a suitable site had been secured long ago, as it should have been, the present scrimmage would never have occurred, and the eleventh hour is too late for action when a fever outbreak occurs.

